# 4-(4-Meth­oxy­phen­yl)-6-methyl­amino-5-nitro-2-phenyl-4*H*-pyran-3-carbonitrile

**DOI:** 10.1107/S1600536813004923

**Published:** 2013-02-28

**Authors:** R. Vishnupriya, J. Suresh, S. Sivakumar, R. Ranjith Kumar, P. L. Nilantha Lakshman

**Affiliations:** a Department of Physics, The Madura College, Madurai 625 011, India; b Department of Organic Chemistry, School of Chemistry, Madurai Kamaraj University, Madurai 625 021, India; c Department of Food Science and Technology, University of Ruhuna, Mapalana, Kamburupitiya 81100, Sri Lanka

## Abstract

In the title compound, C_20_H_17_N_3_O_4_, the central pyran ring adopts a boat conformation with the O atom and diagonally opposite C atoms displaced by 0.1171 (1) and 0.1791 (1) Å, respectively, from the mean plane defined by the other four atoms. The coplanar atoms of the pyran ring and the meth­oxy­benzene ring are nearly perpendicular, as evidenced by the dihedral angle 87.01 (1)°. The amine H atom forms an intra­molecular N—H⋯O(nitro) hydrogen bond. In the crystal, mol­ecules are linked into dimeric aggregates by N—H⋯O(nitro) hydrogen bonds, generating an *R*_2_^2^(12) graph-set motif.

## Related literature

For background to compounds containing the 4*H*-pyran unit, see: Brahmachari (2010 ▶); Hatakeyama *et al.* (1988 ▶). For 2-amino-4*H*-pyrans as photoactive materials, see: Armetso *et al.* (1989 ▶). For graph-set motifs, see: Bernstein *et al.* (1995 ▶). For ring conformation analysis, see: Cremer & Pople (1975 ▶).
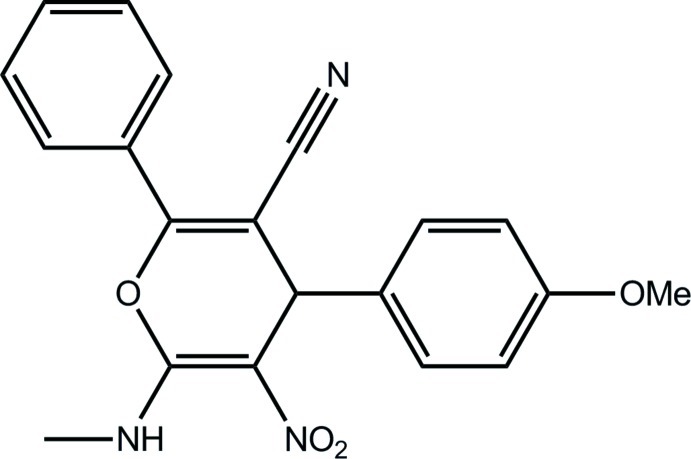


## Experimental

### 

#### Crystal data

C_20_H_17_N_3_O_4_
*M* *_r_* = 363.37
Monoclinic, 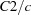

*a* = 22.9422 (10) Å
*b* = 7.5828 (3) Å
*c* = 22.7319 (10) Å
β = 112.576 (2)°
*V* = 3651.5 (3) Å^3^
*Z* = 8
Mo *K*α radiation
μ = 0.09 mm^−1^
*T* = 293 K
0.23 × 0.21 × 0.19 mm

#### Data collection

Bruker Kappa APEXII diffractometer
Absorption correction: multi-scan (*SADABS*; Sheldrick, 1996 ▶) *T*_min_ = 0.967, *T*_max_ = 0.974
15550 measured reflections
4003 independent reflections
2915 reflections with *I* > 2σ(*I*)
*R*_int_ = 0.028

#### Refinement

*R*[*F* ^2^ > 2σ(*F* ^2^)] = 0.040
*wR*(*F* ^2^) = 0.116
*S* = 1.05
4003 reflections
246 parameters
H-atom parameters constrained
Δρ_max_ = 0.23 e Å^−3^
Δρ_min_ = −0.18 e Å^−3^

### 

Data collection: *APEX2* (Bruker, 2004 ▶); cell refinement: *SAINT* (Bruker, 2004 ▶); data reduction: *SAINT*; program(s) used to solve structure: *SHELXS97* (Sheldrick, 2008 ▶); program(s) used to refine structure: *SHELXL97* (Sheldrick, 2008 ▶); molecular graphics: *PLATON* (Spek, 2009 ▶); software used to prepare material for publication: *SHELXL97*.

## Supplementary Material

Crystal structure: contains datablock(s) global, I. DOI: 10.1107/S1600536813004923/tk5197sup1.cif

Structure factors: contains datablock(s) I. DOI: 10.1107/S1600536813004923/tk5197Isup2.hkl

Supplementary material file. DOI: 10.1107/S1600536813004923/tk5197Isup3.cml

Additional supplementary materials:  crystallographic information; 3D view; checkCIF report

## Figures and Tables

**Table 1:** Hydrogen-bond geometry (Å, °)

*D*—H⋯*A*	*D*—H	H⋯*A*	*D*⋯*A*	*D*—H⋯*A*
N2—H2⋯O2	0.86	2.00	2.6203 (19)	128
N2—H2⋯O2^i^	0.86	2.26	3.0114 (18)	147

Symmetry code: (i) 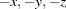.

**Fig. 1.**
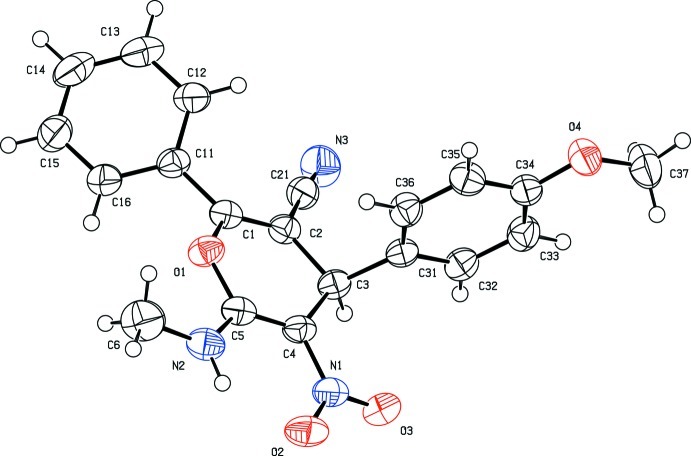
The molecular structure of (I), showing 50% probability displacement ellipsoids and the atom-numbering scheme.

**Fig. 2.**
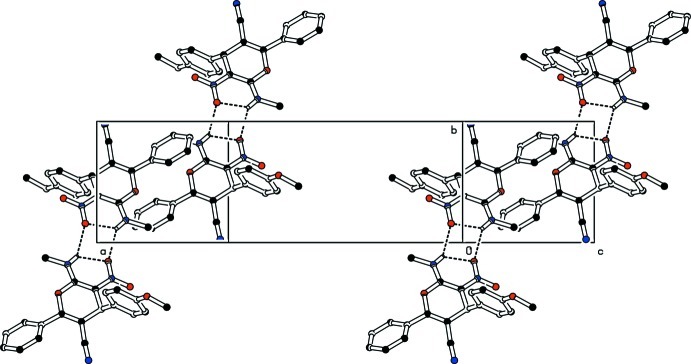
Partial packing diagram showing N—H···O interactions as dashed lines.

## supplementary crystallographic information

### Comment

4*H*-Pyran units constitute structural features of a broad range of bioactive natural products (Brahmachari, 2010; Hatakeyama *et al.*, 1988). 2-Amino-4*H*-pyrans have also been found to be useful as photoactive materials (Armetso *et al.*, 1989). Hence, investigation of the structural features of biologically relevant tetrahydrobenzo[*b*]pyran derivatives is of both scientific and practical interest. In continuation of our efforts to develop useful synthetic protocols for biologically significant molecules, we herein report an efficient and environmentally benign synthesis and the crystal structure of the title compound.

In the title compound, Fig. 1, the six-membered central pyran ring adopts a boat conformation as evidenced by the puckering parameters q_2_ = 0.1713 (16) Å, θ = 98.1 (5)°, φ = 3.5 (6)° (Cremer & Pople, 1975). The dihedral angle between the methoxybenzene ring and the flat part of the pyran ring is 87.01 (1)° which means that the methoxybenzene ring is nearly perpendicular to the pyran ring. The acetonitrile group is almost coplanar with the plane of the pyrazole ring [the N3—C21—C2—C1 torsion angle is 174.04 (16) °]. The nitrile group has a typical bond length, *i.e*. N ≡C = 1.141 (3) Å. The dihedral angle between the flat part of the pyran ring and the phenyl ring is 38.62 (2)°. The phenyl ring is attached to the pyran ring by an (-)-*syn*-clinal conformation with torsion angle C12—C11—C1—C2 of -41.54 (3)°. Similarly, the methoxybenzene ring is attached to the pyran ring by a torsion angle C4—C3—C31—C36 of -59.51 (2)°, again indicating an (-)-*syn*-clinal conformation. The nitro group is attached to pyran ring at C4 with the torsion angle (C5—C4—N1—O2) of -4.89 (3)°, indicating an (-)-*syn*-periplanar conformation.

In the crystal structure, N2—H2···O2 hydrogen bonds link molecules into dimeric pairs, Table 1. Each of these pairs generate a graph set motif of *R*_2_^2^(12) (Bernstein *et al.*, 1995), Fig. 2. In addition, there is a N—H···O intramolecular interaction which stabilizes the structure.

### Experimental

A mixture of benzoylacetonitrile (1.0 mmol), 4-methoxy aldehyde (1.0 mmol), Et_3_N (1.0 mmol) and EtOH (10 ml) were taken in 50 ml round bottom flask. The reaction mixture was stirred at room temperature for 5–10 min. Then *N*-methyl-1-(methylthio)-2-nitroethenamine was added into the reaction mixture followed by refluxing at 353 K. The consumption of starting material was monitored by TLC. After 90 min, the solid product was filtered and washed with diethyl ether (5 ml) and dried under vacuum in 92% yield; *M*.pt: 481 K.

### Refinement

H atoms were placed at calculated positions and allowed to ride on their carrier atoms with C—H = 0.93–0.98 Å and N—H = 0.86 Å, and with *U*_iso_ = 1.2*U*_eq_(C, N) for N, CH_2_ and CH H atoms and *U*_iso_ = 1.5*U*_eq_(C) for CH_3_ H atoms.

### Crystal data

**Table:**

C_20_H_17_N_3_O_4_	*F*(000) = 1520
*M**_r_* = 363.37	*D*_x_ = 1.322 Mg m^−^^3^
Monoclinic, *C*2/*c*	Mo *K*α radiation, λ = 0.71073 Å
Hall symbol: -C 2yc	Cell parameters from 2000 reflections
*a* = 22.9422 (10) Å	θ = 2–31°
*b* = 7.5828 (3) Å	µ = 0.09 mm^−^^1^
*c* = 22.7319 (10) Å	*T* = 293 K
β = 112.576 (2)°	Block, colourless
*V* = 3651.5 (3) Å^3^	0.23 × 0.21 × 0.19 mm
*Z* = 8	

### Data collection

**Table:**

Bruker Kappa APEXII diffractometer	4003 independent reflections
Radiation source: fine-focus sealed tube	2915 reflections with *I* > 2σ(*I*)
Graphite monochromator	*R*_int_ = 0.028
Detector resolution: 0 pixels mm^-1^	θ_max_ = 27.0°, θ_min_ = 1.9°
ω and φ scans	*h* = −29→19
Absorption correction: multi-scan (*SADABS*; Sheldrick, 1996)	*k* = −9→9
*T*_min_ = 0.967, *T*_max_ = 0.974	*l* = −25→29
15550 measured reflections	

### Refinement

**Table:**

Refinement on *F*^2^	Primary atom site location: structure-invariant direct methods
Least-squares matrix: full	Secondary atom site location: difference Fourier map
*R*[*F*^2^ > 2σ(*F*^2^)] = 0.040	Hydrogen site location: inferred from neighbouring sites
*wR*(*F*^2^) = 0.116	H-atom parameters constrained
*S* = 1.05	*w* = 1/[σ^2^(*F*_o_^2^) + (0.0488*P*)^2^ + 2.0393*P*] where *P* = (*F*_o_^2^ + 2*F*_c_^2^)/3
4003 reflections	(Δ/σ)_max_ < 0.001
246 parameters	Δρ_max_ = 0.23 e Å^−^^3^
0 restraints	Δρ_min_ = −0.18 e Å^−^^3^

### Special details

**Table:**

Geometry. All e.s.d.'s (except the e.s.d. in the dihedral angle between two l.s. planes) are estimated using the full covariance matrix. The cell e.s.d.'s are taken into account individually in the estimation of e.s.d.'s in distances, angles and torsion angles; correlations between e.s.d.'s in cell parameters are only used when they are defined by crystal symmetry. An approximate (isotropic) treatment of cell e.s.d.'s is used for estimating e.s.d.'s involving l.s. planes.
Refinement. Refinement of *F*^2^ against ALL reflections. The weighted *R*-factor *wR* and goodness of fit *S* are based on *F*^2^, conventional *R*-factors *R* are based on *F*, with *F* set to zero for negative *F*^2^. The threshold expression of *F*^2^ > σ(*F*^2^) is used only for calculating *R*-factors(gt) *etc*. and is not relevant to the choice of reflections for refinement. *R*-factors based on *F*^2^ are statistically about twice as large as those based on *F*, and *R*- factors based on ALL data will be even larger.

### Fractional atomic coordinates and isotropic or equivalent isotropic displacement parameters (Å^2^)

**Table:**

	*x*	*y*	*z*	*U*_iso_*/*U*_eq_	
O1	−0.07195 (5)	0.42117 (14)	0.09142 (5)	0.0433 (3)	
O2	0.03608 (6)	0.15716 (16)	0.01309 (6)	0.0568 (3)	
O3	0.10675 (6)	0.36536 (17)	0.04434 (6)	0.0552 (3)	
O4	0.26059 (5)	0.44513 (17)	0.34242 (5)	0.0557 (3)	
N1	0.05627 (6)	0.29947 (18)	0.04218 (6)	0.0430 (3)	
N2	−0.06629 (7)	0.18295 (18)	0.03754 (6)	0.0469 (3)	
H2	−0.0477	0.1171	0.0193	0.056*	
N3	0.03447 (8)	0.9733 (2)	0.15792 (8)	0.0609 (4)	
C1	−0.05459 (7)	0.59078 (19)	0.11380 (7)	0.0368 (3)	
C2	0.00202 (7)	0.65426 (19)	0.12158 (6)	0.0365 (3)	
C3	0.05189 (7)	0.54950 (19)	0.10871 (7)	0.0375 (3)	
H3	0.0666	0.6217	0.0814	0.045*	
C4	0.02191 (7)	0.38689 (19)	0.07184 (7)	0.0373 (3)	
C5	−0.03688 (7)	0.3264 (2)	0.06585 (6)	0.0380 (3)	
C6	−0.12770 (10)	0.1270 (3)	0.03463 (11)	0.0686 (6)	
H6A	−0.1358	0.0086	0.0185	0.103*	
H6B	−0.1596	0.2042	0.0069	0.103*	
H6C	−0.1285	0.1310	0.0765	0.103*	
C11	−0.10683 (7)	0.6820 (2)	0.12361 (7)	0.0389 (3)	
C12	−0.09563 (8)	0.7928 (2)	0.17560 (8)	0.0488 (4)	
H12	−0.0549	0.8048	0.2062	0.059*	
C13	−0.14469 (9)	0.8848 (3)	0.18185 (9)	0.0588 (5)	
H13	−0.1370	0.9592	0.2166	0.071*	
C14	−0.20481 (10)	0.8673 (3)	0.13714 (9)	0.0629 (5)	
H14	−0.2376	0.9317	0.1411	0.075*	
C15	−0.21673 (8)	0.7544 (3)	0.08635 (8)	0.0591 (5)	
H15	−0.2577	0.7407	0.0566	0.071*	
C16	−0.16784 (8)	0.6615 (2)	0.07965 (7)	0.0477 (4)	
H16	−0.1760	0.5849	0.0454	0.057*	
C21	0.01831 (7)	0.8329 (2)	0.14170 (7)	0.0421 (4)	
C31	0.10863 (7)	0.51449 (19)	0.17036 (7)	0.0367 (3)	
C32	0.16598 (7)	0.5910 (2)	0.17986 (8)	0.0433 (4)	
H32	0.1700	0.6576	0.1473	0.052*	
C33	0.21774 (7)	0.5718 (2)	0.23641 (8)	0.0469 (4)	
H33	0.2559	0.6256	0.2418	0.056*	
C34	0.21227 (7)	0.4722 (2)	0.28475 (7)	0.0422 (4)	
C35	0.15538 (8)	0.3913 (2)	0.27582 (7)	0.0461 (4)	
H35	0.1517	0.3225	0.3081	0.055*	
C36	0.10402 (7)	0.4126 (2)	0.21914 (7)	0.0438 (4)	
H36	0.0660	0.3581	0.2136	0.053*	
C37	0.31860 (9)	0.5346 (3)	0.35284 (10)	0.0708 (6)	
H37A	0.3487	0.5077	0.3948	0.106*	
H37B	0.3113	0.6595	0.3490	0.106*	
H37C	0.3347	0.4966	0.3217	0.106*	

### Atomic displacement parameters (Å^2^)

**Table:**

	*U*^11^	*U*^22^	*U*^33^	*U*^12^	*U*^13^	*U*^23^
O1	0.0501 (6)	0.0368 (6)	0.0491 (6)	−0.0021 (5)	0.0259 (5)	−0.0061 (5)
O2	0.0772 (9)	0.0453 (7)	0.0536 (7)	0.0048 (6)	0.0315 (6)	−0.0136 (6)
O3	0.0542 (7)	0.0649 (8)	0.0549 (7)	0.0049 (6)	0.0303 (6)	−0.0050 (6)
O4	0.0465 (7)	0.0642 (8)	0.0476 (6)	0.0033 (6)	0.0085 (5)	−0.0063 (6)
N1	0.0529 (8)	0.0424 (8)	0.0351 (6)	0.0088 (6)	0.0184 (6)	0.0007 (6)
N2	0.0588 (9)	0.0369 (7)	0.0464 (7)	−0.0034 (6)	0.0219 (6)	−0.0069 (6)
N3	0.0631 (10)	0.0444 (9)	0.0710 (10)	−0.0037 (8)	0.0209 (8)	−0.0091 (8)
C1	0.0447 (8)	0.0340 (8)	0.0321 (7)	0.0022 (7)	0.0153 (6)	−0.0003 (6)
C2	0.0422 (8)	0.0328 (8)	0.0339 (7)	0.0038 (6)	0.0138 (6)	0.0000 (6)
C3	0.0427 (8)	0.0352 (8)	0.0383 (7)	0.0017 (6)	0.0198 (6)	0.0017 (6)
C4	0.0457 (9)	0.0345 (8)	0.0334 (7)	0.0063 (7)	0.0173 (6)	−0.0008 (6)
C5	0.0502 (9)	0.0322 (8)	0.0324 (7)	0.0049 (7)	0.0167 (6)	0.0014 (6)
C6	0.0754 (14)	0.0569 (12)	0.0797 (13)	−0.0223 (10)	0.0367 (11)	−0.0171 (10)
C11	0.0438 (9)	0.0399 (8)	0.0369 (7)	0.0038 (7)	0.0197 (6)	0.0036 (6)
C12	0.0538 (10)	0.0542 (10)	0.0419 (8)	0.0036 (8)	0.0224 (7)	−0.0048 (7)
C13	0.0724 (13)	0.0603 (12)	0.0570 (10)	0.0090 (10)	0.0396 (10)	−0.0056 (9)
C14	0.0657 (12)	0.0717 (13)	0.0672 (12)	0.0216 (10)	0.0431 (10)	0.0088 (10)
C15	0.0468 (10)	0.0821 (14)	0.0525 (10)	0.0111 (9)	0.0235 (8)	0.0098 (10)
C16	0.0478 (9)	0.0574 (10)	0.0407 (8)	0.0031 (8)	0.0200 (7)	−0.0001 (7)
C21	0.0414 (8)	0.0419 (9)	0.0416 (8)	0.0046 (7)	0.0143 (7)	−0.0002 (7)
C31	0.0409 (8)	0.0323 (8)	0.0394 (7)	0.0032 (6)	0.0182 (6)	−0.0037 (6)
C32	0.0443 (9)	0.0421 (9)	0.0483 (8)	−0.0010 (7)	0.0232 (7)	0.0010 (7)
C33	0.0404 (9)	0.0464 (10)	0.0557 (9)	−0.0053 (7)	0.0203 (7)	−0.0052 (8)
C34	0.0411 (8)	0.0397 (9)	0.0434 (8)	0.0050 (7)	0.0136 (7)	−0.0087 (7)
C35	0.0517 (10)	0.0457 (9)	0.0420 (8)	−0.0007 (8)	0.0192 (7)	0.0042 (7)
C36	0.0414 (9)	0.0447 (9)	0.0470 (9)	−0.0054 (7)	0.0190 (7)	0.0019 (7)
C37	0.0469 (11)	0.0847 (15)	0.0647 (12)	−0.0051 (10)	0.0034 (9)	−0.0130 (11)

### Geometric parameters (Å, º)

**Table:**

O1—C5	1.3641 (18)	C11—C12	1.391 (2)
O1—C1	1.3842 (18)	C12—C13	1.377 (2)
O2—N1	1.2573 (17)	C12—H12	0.9300
O3—N1	1.2448 (17)	C13—C14	1.370 (3)
O4—C34	1.3688 (19)	C13—H13	0.9300
O4—C37	1.430 (2)	C14—C15	1.378 (3)
N1—C4	1.3868 (18)	C14—H14	0.9300
N2—C5	1.311 (2)	C15—C16	1.382 (2)
N2—C6	1.448 (2)	C15—H15	0.9300
N2—H2	0.8602	C16—H16	0.9300
N3—C21	1.141 (2)	C31—C32	1.377 (2)
C1—C2	1.332 (2)	C31—C36	1.388 (2)
C1—C11	1.473 (2)	C32—C33	1.383 (2)
C2—C21	1.432 (2)	C32—H32	0.9300
C2—C3	1.511 (2)	C33—C34	1.378 (2)
C3—C4	1.501 (2)	C33—H33	0.9300
C3—C31	1.526 (2)	C34—C35	1.385 (2)
C3—H3	0.9800	C35—C36	1.382 (2)
C4—C5	1.381 (2)	C35—H35	0.9300
C6—H6A	0.9600	C36—H36	0.9300
C6—H6B	0.9600	C37—H37A	0.9600
C6—H6C	0.9600	C37—H37B	0.9600
C11—C16	1.381 (2)	C37—H37C	0.9600
			
C5—O1—C1	120.81 (12)	C14—C13—C12	120.29 (17)
C34—O4—C37	116.60 (15)	C14—C13—H13	119.9
O3—N1—O2	120.97 (13)	C12—C13—H13	119.9
O3—N1—C4	118.86 (13)	C13—C14—C15	120.09 (16)
O2—N1—C4	120.17 (14)	C13—C14—H14	120.0
C5—N2—C6	125.02 (15)	C15—C14—H14	120.0
C5—N2—H2	117.5	C14—C15—C16	120.06 (17)
C6—N2—H2	117.5	C14—C15—H15	120.0
C2—C1—O1	120.88 (13)	C16—C15—H15	120.0
C2—C1—C11	128.23 (14)	C11—C16—C15	120.19 (16)
O1—C1—C11	110.85 (12)	C11—C16—H16	119.9
C1—C2—C21	120.38 (14)	C15—C16—H16	119.9
C1—C2—C3	123.82 (13)	N3—C21—C2	176.34 (17)
C21—C2—C3	115.77 (13)	C32—C31—C36	118.06 (14)
C4—C3—C2	108.74 (12)	C32—C31—C3	119.84 (13)
C4—C3—C31	114.61 (12)	C36—C31—C3	122.05 (13)
C2—C3—C31	110.87 (11)	C31—C32—C33	121.95 (15)
C4—C3—H3	107.4	C31—C32—H32	119.0
C2—C3—H3	107.4	C33—C32—H32	119.0
C31—C3—H3	107.4	C34—C33—C32	119.37 (15)
C5—C4—N1	120.62 (14)	C34—C33—H33	120.3
C5—C4—C3	123.27 (13)	C32—C33—H33	120.3
N1—C4—C3	116.10 (13)	O4—C34—C33	123.88 (15)
N2—C5—O1	111.68 (14)	O4—C34—C35	116.45 (15)
N2—C5—C4	128.53 (14)	C33—C34—C35	119.67 (14)
O1—C5—C4	119.79 (13)	C36—C35—C34	120.22 (15)
N2—C6—H6A	109.5	C36—C35—H35	119.9
N2—C6—H6B	109.5	C34—C35—H35	119.9
H6A—C6—H6B	109.5	C35—C36—C31	120.72 (15)
N2—C6—H6C	109.5	C35—C36—H36	119.6
H6A—C6—H6C	109.5	C31—C36—H36	119.6
H6B—C6—H6C	109.5	O4—C37—H37A	109.5
C16—C11—C12	119.20 (14)	O4—C37—H37B	109.5
C16—C11—C1	119.67 (14)	H37A—C37—H37B	109.5
C12—C11—C1	121.10 (14)	O4—C37—H37C	109.5
C13—C12—C11	120.12 (16)	H37A—C37—H37C	109.5
C13—C12—H12	119.9	H37B—C37—H37C	109.5
C11—C12—H12	119.9		
			
C5—O1—C1—C2	−12.1 (2)	C2—C1—C11—C12	−41.5 (2)
C5—O1—C1—C11	165.46 (12)	O1—C1—C11—C12	141.09 (14)
O1—C1—C2—C21	176.84 (13)	C16—C11—C12—C13	−2.0 (2)
C11—C1—C2—C21	−0.3 (2)	C1—C11—C12—C13	176.20 (15)
O1—C1—C2—C3	−1.5 (2)	C11—C12—C13—C14	0.3 (3)
C11—C1—C2—C3	−178.63 (13)	C12—C13—C14—C15	1.4 (3)
C1—C2—C3—C4	13.69 (19)	C13—C14—C15—C16	−1.4 (3)
C21—C2—C3—C4	−164.72 (12)	C12—C11—C16—C15	2.0 (2)
C1—C2—C3—C31	−113.18 (15)	C1—C11—C16—C15	−176.22 (15)
C21—C2—C3—C31	68.41 (16)	C14—C15—C16—C11	−0.3 (3)
O3—N1—C4—C5	174.32 (13)	C1—C2—C21—N3	174 (100)
O2—N1—C4—C5	−4.9 (2)	C3—C2—C21—N3	−7 (3)
O3—N1—C4—C3	−4.48 (19)	C4—C3—C31—C32	123.21 (15)
O2—N1—C4—C3	176.31 (12)	C2—C3—C31—C32	−113.23 (15)
C2—C3—C4—C5	−14.61 (19)	C4—C3—C31—C36	−59.51 (18)
C31—C3—C4—C5	110.08 (16)	C2—C3—C31—C36	64.05 (18)
C2—C3—C4—N1	164.15 (12)	C36—C31—C32—C33	−1.3 (2)
C31—C3—C4—N1	−71.16 (16)	C3—C31—C32—C33	176.07 (14)
C6—N2—C5—O1	−1.5 (2)	C31—C32—C33—C34	0.5 (2)
C6—N2—C5—C4	178.56 (17)	C37—O4—C34—C33	3.3 (2)
C1—O1—C5—N2	−168.93 (12)	C37—O4—C34—C35	−177.38 (15)
C1—O1—C5—C4	11.0 (2)	C32—C33—C34—O4	180.00 (14)
N1—C4—C5—N2	4.7 (2)	C32—C33—C34—C35	0.7 (2)
C3—C4—C5—N2	−176.58 (14)	O4—C34—C35—C36	179.60 (14)
N1—C4—C5—O1	−175.20 (12)	C33—C34—C35—C36	−1.0 (2)
C3—C4—C5—O1	3.5 (2)	C34—C35—C36—C31	0.2 (2)
C2—C1—C11—C16	136.64 (17)	C32—C31—C36—C35	1.0 (2)
O1—C1—C11—C16	−40.72 (18)	C3—C31—C36—C35	−176.37 (14)

### Hydrogen-bond geometry (Å, º)

**Table:**

*D*—H···*A*	*D*—H	H···*A*	*D*···*A*	*D*—H···*A*
N2—H2···O2	0.86	2.00	2.6203 (19)	128
N2—H2···O2^i^	0.86	2.26	3.0114 (18)	147

Symmetry code: (i) −*x*, −*y*, −*z*.
